# The Translational Role of miRNA in Polycystic Ovary Syndrome: From Bench to Bedside—A Systematic Literature Review

**DOI:** 10.3390/biomedicines10081816

**Published:** 2022-07-28

**Authors:** Salvatore Giovanni Vitale, Anna Maria Fulghesu, Mislav Mikuš, Rafał Watrowski, Maurizio Nicola D’Alterio, Li-Te Lin, Mohsin Shah, Enrique Reyes-Muñoz, Thozhukat Sathyapalan, Stefano Angioni

**Affiliations:** 1 Obstetrics and Gynecology Unit, Department of General Surgery and Medical Surgical Specialties, University of Catania, 95124 Catania, Italy; sgvitale@unict.it; 2 Division of Gynecology and Obstetrics, Department of Surgical Sciences, University of Cagliari, 09124 Cagliari, Italy; amfulghesu@unica.it (A.M.F.); mauridalte84@gmail.com (M.N.D.); 3 Department of Obstetrics and Gynecology, University Hospital Centre Zagreb, 10 000 Zagreb, Croatia; m.mikus19@gmail.com; 4 Faculty of Medicine, University of Freiburg, 79106 Freiburg, Germany; rafal.watrowski@gmx.at; 5 Department of Obstetrics and Gynecology, Kaohsiung Veterans General Hospital, Kaohsiung City 81362, Taiwan; litelin1982@gmail.com; 6 Department of Obstetrics and Gynecology, School of Medicine, National Yang-Ming University, Pei-Tou, Taipei 112, Taiwan; 7 Department of Biological Science, National Sun Yat-sen University, 70 Lienhai Rd., Kaohsiung City 80424, Taiwan; 8 Department of Physiology, Institute of Basic Medical Sciences, Khyber Medical University, Peshawar 25100, Pakistan; mohsin.ibms@kmu.edu.pk; 9 Department of Gynecological and Perinatal Endocrinology, Instituto Nacional de Perinatología, Mexico City 11000, Mexico; dr.enriquereyes@gmail.com; 10 Academic Diabetes, Endocrinology and Metabolism, Hull York Medical School, University of Hull, Kingston upon Hull HU6 7RX, UK; thozhukat.sathyapalan@hyms.ac.uk

**Keywords:** microRNA, miRNA, polycystic ovary syndrome, PCOS, hyperandrogenemia, insulin resistance, granulosa cells, theca cells

## Abstract

MicroRNAs (miRNAs) are small, non-coding RNAs that are essential for the regulation of post-transcriptional gene expression during tissue development and differentiation. They are involved in the regulation of manifold metabolic and hormonal processes and, within the female reproductive tract, in oocyte maturation and folliculogenesis. Altered miRNA levels have been observed in oncological and inflammatory diseases, diabetes or polycystic ovary syndrome (PCOS). Therefore, miRNAs are proving to be promising potential biomarkers. In women with PCOS, circulating miRNAs can be obtained from whole blood, serum, plasma, urine, and follicular fluid. Our systematic review summarizes data from 2010–2021 on miRNA expression in granulosa and theca cells; the relationship between miRNAs, hormonal changes, glucose and lipid metabolism in women with PCOS; and the potential role of altered miRNAs in fertility (oocyte quality) in PCOS. Furthermore, we discuss miRNAs as a potential therapeutic target in PCOS and as a diagnostic marker for PCOS.

## 1. Introduction

MicroRNAs (miRNAs) are short, single-stranded, non-coding and evolutionarily highly conserved RNA molecules that are found in eukaryotic cells and are responsible for controlling gene expression at the post-translational and transcriptional levels [1]. MiRNAs play a significant role in the regulation of signaling pathways and basic cellular processes such as proliferation and differentiation, and they play an important role in neuronal development and function, immunity and the maintenance of homeostasis through the regulation of programmed cell death [2,3]. RNA polymerase II is one of the key enzymes for the transcription of miRNA into the initial long stem-loop structure of primary miRNA (pri-miRNA) [4]. The enzyme binds to a DNA sequence promoter that encodes a loop to form a newly formed miRNA molecule. In the nucleus, the pri-miRNA is cleaved by the enzyme Drosha, resulting in a hairpin-structured pre-miRNA that can be transported into the cytoplasm via the exportin-5 complex for further maturation [5,6]. The enzyme Dicer-TRBP (TAR-RNA binding protein) removes the loop of the pre-miRNA molecule—what remains is a short nucleotide duplex consisting of a lead chain and a passenger chain [7,8]. A chain with a thermodynamically more stable 5′ end (“passenger” chain) is degraded by an unknown mechanism, while the lead chain is formed into a mature miRNA that binds to one of the proteins from the Argonaute family (AGO 1–4 in humans) to form the RNA-induced silencing complex (RISC) [9]. As part of the RISC complex, mature miRNAs can regulate gene expression by recognizing the corresponding mRNA. After recognition and due to the endonuclease activity of the AGO family proteins, the degradation of the mRNA or inhibition of its translation occurs depending on the degree of complementarity [10,11].

Several studies from the last two decades have demonstrated a clear influence of altered miRNA expression on the development of various diseases, including some neurological and autoimmune disorders [12,13,14]; congenital heart diseases such as tetralogy of Fallot [15]; and female reproductive diseases, such as preeclampsia, uterine leiomyomas, endometriosis and polycystic ovary syndrome (PCOS) [16]. Although PCOS manifests itself clinically in a very heterogeneous way, three symptoms predominate increased androgen production, ovulation disorders and ultrasound findings of polycystic ovaries [17,18]. The different diagnostic criteria currently used in clinical practice are heterogenous [19,20,21]. The incidence of PCOS depends on the observed diagnostic criterion (varying between 6.5% and 22%). It is increased in people with obesity, insulin resistance, type 1 or 2 diabetes, anovulatory infertility, premature adrenarche and in those with a close relative with PCOS [22].

PCOS is a common endocrine-metabolic disorder that carries a number of serious consequences for women’s health, including alarming rates of infertility [22,23]. It is denoted by multiple hormonal variations reflected in a clinical picture of hyperandrogenism that has both short- and long-term repercussion for women’s health [24]. The manifestations of PCOS are not only gynecologic in nature, as these patients have an increased prevalence of several, mainly metabolic, comorbidities, including type 2 diabetes, obesity, arterial hypertension, dyslipidemia and metabolic syndrome [25,26]. These features, together with other alterations such as endothelial dysfunction and chronic mild inflammation, lead to an increased risk of cardiovascular disease and all-cause mortality [27].

Despite the clinical and biochemical determinants of PCOS status, the etiology of this syndrome is still unclear. There is increasing evidence that altered miRNA expression may play an important role in the initiation and development of PCOS. Therefore, the aim of this systematic review is to cover all current evidence on miRNA expression patterns and the development of PCOS and to provide insight into the diagnostic potential of circulating miRNAs.

## 2. Materials and Methods

**Figure 1:**
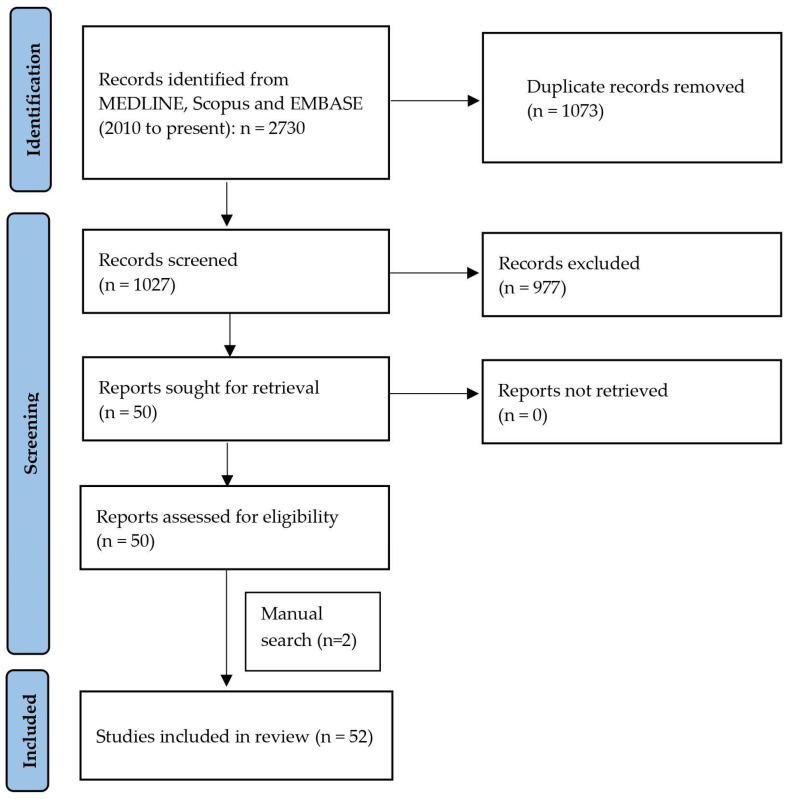
PRISMA flow diagram of the systematic literature search.

We identified relevant original studies in English language through a search of the MEDLINE, Scopus and EMBASE (2010 to present) databases using the following terms: (“miRNA” or “miR”) AND (‘’polycystic ovary’’ or “polycystic ovary syndrome” or “hyperandrogenism” or “infertility” or “anovulation”). The systematic review was made in accordance with the Preferred Reporting Items for Systematic Reviews and Meta-Analyses (PRISMA) guidelines (http://prisma-statement.org/prismastatement/Checklist.aspx (accessed on 26 May 2022). A flow chart of the systematic literature search according to PRISMA guidelines is reported in Figure 1.

Three authors (S.G.V., M.M. and S.A.) independently screened the titles and abstracts of studies obtained by the search strategy. The full text of each potentially relevant study was obtained and assessed for inclusion independently by the two authors (M.M. and S.G.V.). They also independently extracted data from the included studies. Three other authors (R.W., A.M.F. and T.S.) independently reviewed the selection and data extraction process. In this systematic review, we included different types of studies whose results are presented as relative risk (RR) or an odds ratio (OR) with 95% accuracy. The results of studies in which the size and characteristics of the observed groups have not been stated were excluded. Regarding type of research, we included randomized clinical studies, observational studies, retrospective and prospective studies, and cross-sectional and case-control studies only in the English language. Studies without original data, including reviews, comments, editorials and meta-analyses, were not included. The methodological quality of the studies was independently assessed by two investigators (S.G.V. and M.M.) using the nine-star Newcastle–Ottawa scale (NOS), which is a standardized scale for assessing the bias and quality of non-randomized studies.

The results were compared, and any disagreement was discussed and resolved by consensus. Studies providing ambiguous or insufficient, low-quality data or non-quantifiable outcomes were also excluded.

## 3. Results

### 3.1. Literature Search

The electronic searches, after duplicate records removal, provided a total of 1027 citations. Of these, 975 were excluded after title/abstract screening (not relevant to the review inclusion criteria). We examined the full text of 52 publications to summarize the possible translational role of miRNA in PCOS [28,29,30,31,32,33,34,35,36,37,38,39,40,41,42,43,44,45,46,47,48,49,50,51,52,53,54,55,56,57,58,59,60,61,62,63,64,65,66,67,68,69,70,71,72,73,74,75,76,77,78,79]. According to the literature reviewed, our synthesis of results provides information about miRNA expression in granulosa and theca cells; the relationship between miRNAs, glucose and lipid metabolism in PCOS; and the possible role of altered miRNAs in fertility (oocyte quality) in PCOS.

### 3.2. Study Characteristics and Quality

Table 1 shows the detailed characteristics of the included studies after the literature search. The vast majority of studies are originating from China and are case-control studies. Sample size varies from 6 to 372 patients, and the main results are summarized in Table 1 as dysregulated miRNAs detected in granulosa cells, adipose tissue, serum, whole blood, plasma and ovarian tissue. Regarding the main results from the included studies, Table 2 presents a summary of the reported biological role of the most notable dysregulated miRNAs in terms of hormonal homeostasis. The risk of bias among the 52 studies was generally low (around 7 and more points) according to the nine-star NOS scale (Table 3).

## 4. Discussion

### 4.1. PCOS: Definition, Clinical Presentation and Heterogeneity

PCOS is a complex endocrine disorder, firstly described in 1935 by physicians Stein and Leventhal, who cited amenorrhea, polycystic ovaries, and hirsutism as the main clinical features of the syndrome [84]. However, PCOS is a heterogeneous disease that manifests itself in a very wide range of symptoms and features, so different definitions of the disease have been proposed [23]. However, there is still no unified position regarding the nomenclature and criteria used in defining this syndrome. Today, three types of diagnostic criteria for PCOS are in use [19,20,21].

In 1990, the National Institutes of Health defined criteria for diagnosing PCOS that included chronic anovulation and clinical or biochemical signs of hyperandrogenism that were not caused by a disease of other etiology.

The Rotterdam criteria, adopted in 2003, define PCOS as the presence of oligo/anovulation, clinical or biochemical signs of elevated androgen levels, and polycystic ovaries detected by ultrasound. At least two of the above three criteria are required to diagnose PCOS [20]. In 2009, the AES (Androgen Excess Society) proposed a definition of PCOS that includes the presence of elevated androgen levels (hyperandrogenemia and/or hirsutism) and ovarian dysfunction (oligo-anovulation and polycystic ovaries) and excludes conditions of other etiologies. The heterogeneity of the clinical picture of PCOS is best reflected in the Rotterdam criteria [20]. Patients diagnosed with PCOS may belong to four phenotypic groups: Phenotype A (hyperandrogenism, oligo- or anovulation and PCOM), Phenotype B (hyperandrogenism and oligo- or anovulation), Phenotype C (hyperandrogenism and PCOM) or Phenotype D (oligo- or anovulation and PCOM). The phenotypes described represent a wide range of clinical pictures from “classic PCOS” (phenotype A and B) to “ovulatory PCOS” (phenotype C) and “non-hyperandrogenic PCOS” (phenotype D). It is important to emphasise that phenotype affiliation may change throughout life [85].

### 4.2. Metabolic and Fertility Consequences of PCOS

**Figure 2:**
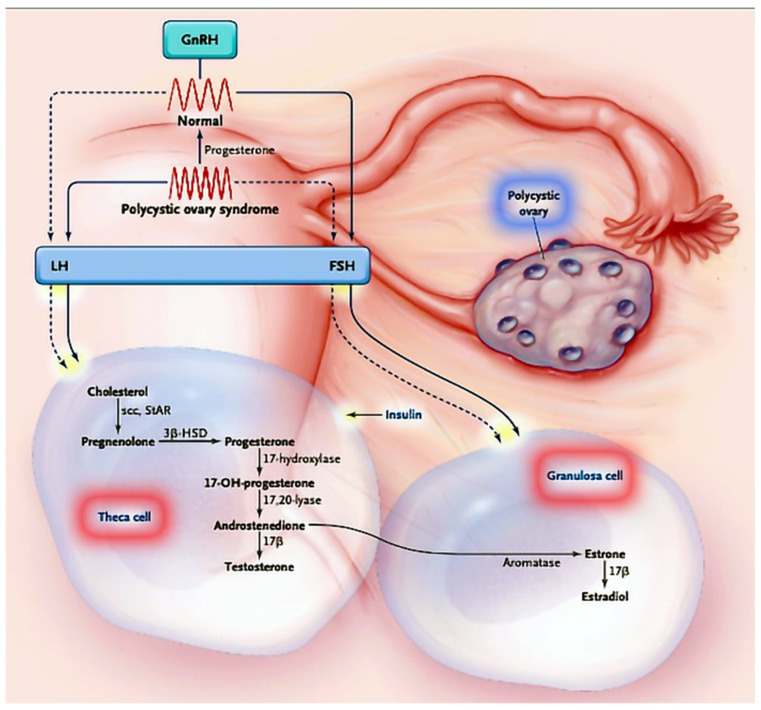
Classical representation of the interplay between hypothalamic, hypophyseal and ovarian hormones and insulin in PCOS (adapted from [88] with permission of the author).

The metabolic consequences of PCOS are mainly linked to insulin resistance and are reflected in glucose and lipid metabolism [86]. Since theca cells, granulosa cells and ovarian tissue stromal cells possess INSR and IGF-1 receptors, this clearly indicates that the ovaries are also the target tissue of insulin action, and this is confirmed by the finding of decreased steroidogenesis in theca and granulosa cells in both healthy and polycystic ovaries [24,86]. One of the major links in this activity is the protein steroidogenesis regulator (StAR), which is important for synthesizing steroid hormones. Insulin increases the expression of StAR but also of 17-α-hydroxylase/17,20-lyase, 3-β-hydroxysteroid dehydrogenase and aromatase, leading to the excessive production of progesterone, 17-α hydroxyprogesterone and testosterone in polycystic ovaries compared with healthy ovaries (Figure 2) [87].

The main paradox in the pathophysiological link between hyperandrogenemia and hyperinsulinemia in PCOS is the ovarian susceptibility to insulin activity (leading to androgen production) despite systemic insulin resistance. For this reason, the theory of ‘selective insulin resistance’ has been developed [24]. Several mechanisms have been proposed to explain this phenomenon, but the correct chain of related processes is still scientifically unattainable.

Although various types of dyslipidemia occur in PCOS, it is most commonly associated with decreased HDL cholesterol and increased circulating triglycerides—a lipid profile known to be associated with insulin resistance [89]. Obesity is also associated with impaired lipoprotein lipase-mediated lipolysis. The tendency for the centripetal placement of adipose tissue in PCOS further contributes to the unfavorable lipid profile [90]. Hyperandrogenism is known to favor the centripetal distribution of adipose tissue. It may further affect the lipid profile by increasing the degradation of HDL cholesterol, decreasing the degradation of LDL and VLDL cholesterols and altering lipoprotein lipase activity [91]. Although hyperandrogenism and disorders of lipid metabolism are closely associated with PCOS, the pathophysiological background of this association and the relationship between hyperandrogenism and cardiovascular disease are not entirely clear.

From the above, it can be concluded that insulin resistance and compensatory hyperinsulinemia, together with hyperandrogenemia and obesity, are the main metabolic disorders leading to dyslipidemia in PCOS.

Strong correlations exist between the metabolic consequences of PCOS and infertility [86,92,93]. Women with PCOS have a 30–50% risk of miscarriage, three times that of normal women, while infertility prevalence in this population is up to 75% [94]. The mechanisms likely involved in the development of miscarriage in these women are: the overexpression of androgen and steroid receptors and the concomitant decreased expression of the molecules of implantation, such as α vs. β3 integrin and glycodelin [95]; hyperinsulinemia, which inhibits endometrial and stromal differentiation in vitro (decidualization) and downregulates IGFBP-1 locally [96]; hypofibrinolysis, mediated by high levels of plasminogen activator inhibitor (PAI) [97]; the increased resistance of uterine artery blood flow leading to reduced subendometrial and endometrial vascularization [98].

It is known that about 85% of women with PCOS have hyperandrogenemia and hyperandrogenism [23]. Elevated androgens originate not only from the ovaries but also from adipose tissue, and, in 20% of patients, androgens from the adrenal gland are also elevated [18]. In patients with PCOS, the selection of the dominant follicle leading to ovulation is impaired. The intraovarian inhibitors of FSH action are responsible for the impaired effect of FSH on aromatase enzyme activity in the granulosa cells of small follicles in the ovary [17,18]. Due to the weaker activity of aromatase, the follicle remains in an unfavorable environment of dominant androgens, which prevents the maturation of the oocyte and leads to hyperandrogenemia and anovulation. The goal of treating anovulatory infertility due to PCOS is to achieve monoovulation and thus achieve a singleton pregnancy. One should always keep in mind that the pregnancy of a woman with PCOS is a serious health risk for both mother and child due to the higher frequency of gestational diabetes, hypertension, eclampsia, premature birth and the birth of children with low birth weight and that these children have increased metabolic risks later in life [94,95,96,97,98].

### 4.3. MicroRNAs in Granulosa Cells

Granulosa cells (GC) play a crucial role not only in normal folliculogenesis but also in pathological folliculogenesis, both in benign diseases such as PCOS and premature ovarian failure (POI) and in malignant diseases such as ovarian granulosa cell tumors. PCOS research models have revealed augmented GC proliferation and increased follicle numbers [40,99]. Hence, it is credible that altered ovarian GC proliferation takes part in the PCOS pathogenesis, however, with undetermined underlying mechanism.

As mentioned previously, Dicer-TRBP is required for miRNA biogenesis. The investigation of the loss of function of Dicer-TRBP in GC sheds light on the role of miRNAs in GC. Otsuka and associates are among the pioneers of demonstrating that a reduction in Dicer-TRBP expression by a hypomorphic mutation leads to corpus luteum insufficiency and defected ovarian angiogenesis [100]. Furthermore, another study found that the loss of Dicer-TRBP function in conditional knockout models leads to several reproductive issues, including impaired oocyte and embryo quality and integrity, diminished ovulation rates and shorter uterine horns [101]. In the GC-specific Dicer-TRBP knockout mice, transport into the fallopian tube was impaired, as evidenced by the inability of embryos to enter the uterus [102]. These studies suggest that Dicer/miRNA-mediated post-transcriptional gene regulation in reproductive somatic tissues is crucial for the appropriate development and function of these tissues and overall female fertility.

There is evidence of the dysregulation of miRNAs in the GCs of PCOS women that can be used to determine potential therapeutic targets [103]. For example, hyperandrogenic PCOS patients (compared with normoandrogenic ones) have been found to have increased expression of miR-21 and miR-93 [53]. Free testosterone and free androgen index positively correlate with miR-21 and miR-93 in PCOS GCs [53]. Since miR-93 and miR-21 were highlighted as androgen-dependent factors, they may play a role in follicular dysfunction involved in the pathogenesis of PCOS under hyperandrogenic conditions. Furthermore, the downregulation of miR-320a GCs is thought to impact relative estrogen deficiency and IGF-1 regulatory mechanisms in PCOS patients [104]. A similar effect on relative estrogen deficiency was associated with miR-27a-3p, which additionally induces GC apoptosis and may also be involved in the pathophysiology of PCOS [105]. By targeting insulin receptor substrate-1 (IRS1) in the ovarian GC of PCOS patients, miR-145 has been linked to the negative regulation of cell proliferation by suppressing the MAPK/ERK signaling pathways [28]. Targeting this pathway should certainly be one of the mechanisms to promote estradiol secretion in the GCs of PCOS patients. Another pathway involved is PI3k/Akt, which is induced by miR-486-5p, fostering the GC proliferation in women with PCOS [81]. MiR-93 expression is upregulated in PCOS-GCs, and its predicted target, cyclin-dependent kinase inhibitor 1A (CDKN1A), is suppressed in PCOS-GCs [42]. Therefore, miR-93 promotes ovarian GC proliferation by targeting CDKN1A in PCOS.

There is sufficient evidence to support a link between miRNA dysregulation in GCs and PCOS based on published studies. This suggests that miRNA manipulation may play a credible role in improving follicular health in women with PCOS.

### 4.4. MicroRNAs in Theca Cells

Studies have shown that miRNAs also play an important role in ovarian theca cell function [106,107]. Both GCs and theca cells produce (non)steroidal factors that influence mutual proliferation and differentiation during folliculogenesis. Lin and associates, in their case-control study, reported that the expression of miR-92a and miR-92b was significantly downregulated in the theca cells of women with PCOS [41]. Moreover, studies indicated that GATA6 and IRS-2 were significantly higher in PCOS theca cells [108,109,110]. GATA6 is an important androgen-production-related gene whose protein has been shown to stimulate the human CYP17 promoter activity [111]. IRS-2 is an insulin receptor gene whose products lead to increased PI3K activity and could further promote androgen production by regulating the activity of thecal 17α-hydroxylase [112]. Therefore, miR-92a and miR-92b downregulation may play a role in the androgen biosynthesis dysregulation in theca cells of women with PCOS.

Notably, the data on miRNAs in theca cells in PCOS are lacking, but published studies indicate that the dysregulated miRNAs in ovarian theca cells may be involved in hyperandrogenemia and PCOS evolution.

### 4.5. MicroRNAs and Fertility in PCOS

Despite the inconsistencies between studies, dysregulated miRNAs in follicular fluid may be one of the key factors in the ovulatory dysfunction of women with PCOS [80,113]. Several studies have pointed to the importance of the downregulation of miR-202-5p and the subsequent dysregulation of the SLIT-ROBO pathway, one of the crucial steps in disrupted cellular communication in the ovarian tissue [114]. This role was confirmed by the study of Gay and colleagues, in which the knockout of the miR-202-5p gene in medaka ovaries reduced the expression of the Gdf and Foxl2b genes, resulting in either no oogenesis or the reduced production of oocytes that later fail to mature and fertilize [82]. The recently published prospective study by Khan and coworkers examined the expression profiles of several extracellular miRNAs in human follicular fluid with the aim of investigating the outcomes of the IVF/ICSI [115]. They examined a panel of several differentially expressed miRNAs (miR-7-5p, miR-202-5p, miR-378-3p, miR-224, miR-320a, miRNA-212-3p and miR-21-5p) that successfully discriminated between women with normoandrogenic PCOS and women with normal ovarian tests. This panel has shown a sensitivity of 79.2% and a specificity of 87.32%. (AUC = 0.881 [0.61; 0.92], *p* = 0.001). Another important finding of this study is that the overexpression of miR-378-3p may be one of the causes of the relatively high density of the primary follicular population in PCOS patients.

There is not yet sufficient and convincing evidence regarding the role of dysregulated miRNA in assisted reproductive technology. The results of Khan et al. show the significantly differential expression of miR-320a in best-quality embryos compared with nonbest-quality embryos, with a sensitivity of 80% and a specificity of 71% (AUC = [0.753 (0.651; 0.855)], *p* = 0.001) [115]. Although there is growing evidence for the direct contribution of an altered miRNA profile and the potential for tailored IVF treatment strategies in the management of infertility, endometrial receptivity remains a limiting step for the overall success of IVF in humans.

### 4.6. MicroRNAs, Glucose and Lipid Metabolism in PCOS

**Figure 3:**
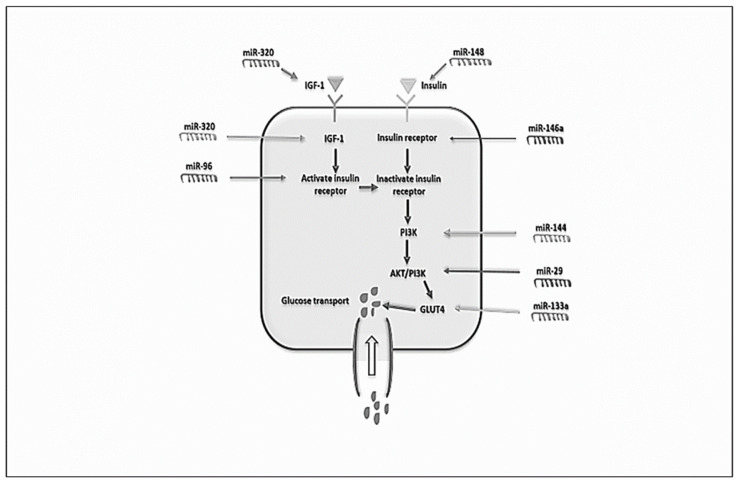
Role of miRNAs in the insulin signaling pathway and resistance (adapted from Abdalla et al., 2020 [119] with kind permission of Elsevier).

Inherent insulin resistance is present in approximately 60–70% of women with PCOS [116,117]. This condition leads to compensatory hyperinsulinemia and an increased risk of diabetes mellitus type 2 and cardiovascular disease [117]. The underlying cellular mechanisms that cause insulin resistance originate primarily in subcutaneous adipose tissue and may include decreased GLUT4 protein expression and production, interrupted glucose transport and impaired lipolysis [118]. Since we have already mentioned the importance of altered miRNA expression in PCOS pathogenesis, miRNAs also affect many cellular functions, including glucose and lipid metabolism. Figure 3 illustrates the important role of miRNAs in the insulin signaling pathway and in insulin resistance.

Differential miRNA expression has also been found in insulin-resistant adipocytes [120]. The role of GLUT4 in glucose metabolism and glucose tolerance is to facilitate insulin-mediated glucose transport into adipocytes to ensure adequate glucose homeostasis [121]. GLUT4 expression in cardiac myocytes is modulated by miRNAs. miR-133 was found to regulate GLUT4 expression by targeting KLF15, and the overexpression of miR-223 increased GLUT4 expression and enhanced the glucose uptake [122]. In PCOS patients, GLUT4 protein expression is significantly decreased; thus, this may play an essential part in insulin resistance in those patients. Another study confirmed this observation and investigated the role of miRNAs in the GLUT4 regulation in subcutaneous adipose tissue of PCOS patients and comparable control subjects [29]. The authors found altered miRNA expression in PCOS patients, including the upregulation of miR-93, miR-133 and miR-223, and miR-93 was also found to be upregulated in control subjects with insulin resistance [29]. Although a study by Ciaraldi and colleagues found no difference in GLUT4 levels between PCOS women and control subjects [123], there is a consensus that the regulation of GLUT4 expression is a critical molecular endpoint for insulin resistance in the adipose tissue of PCOS patients.

Interestingly, many miRNAs, such as miR-123, miR-143 and miR-144, were overexpressed in the adipose tissue of lean PCOS women, indicating the role of miRNAs in insulin signaling [124]. The current scientific and practical focus is on miR-93, as it was overexpressed in the adipose tissue of PCOS patients and is thought to target the 3′UTR of GLUT4, indicating its central role in insulin-mediated glucose regulation. Some authors observed that PCOS patients have intrinsic overexpression of miR-93, independent of BMI [29]. PCOS is associated with obesity and impaired lipid metabolism. Obesity is pandemic in the PCOS population—up to 85% of women with PCOS are at least overweight [125]. Although a link between PCOS and all metabolic consequences of obesity is proven and obvious, there is still insufficient data on the role of miRNAs in dyslipidemia in PCOS patients. In mouse liver and cultured hepatocytes, the overexpression of miR-451a was associated with a reduction in triglyceride accumulation [126]. miR-33 has been shown to target adenosine triphosphate (ATP)-binding cascade transporter A1 (ABCA1), an important regulator that increases HDL-C levels and facilitates cholesterol breakdown in the liver [119]. Furthermore, miR-122 and miR-30c play essential roles in controlling LDL-C by regulating cholesterol biosynthesis and VLDL-C secretion [127]. Targeting miR-122 in an animal model has shown to result in a significant reduction in cholesterol and triglyceride levels. However, this effect was overshadowed by the increased risk of liver cancer and fibrosis associated with the deletion of miR-122 [83]. Arancio and coworkers showed that the serum levels of miR-320 and miR-30a-3p were inversely correlated with LDL cholesterol concentration in women with hyperandrogenic PCOS [128]. Therefore, these results indicate that miRNA may be a promising target for regulating lipid metabolism in PCOS.

### 4.7. Future Directions: miRNAs as a Potential Therapeutic Target in PCOS and as a Diagnostic Marker for PCOS

Although there is still no unified position regarding the nomenclature and criteria to define PCOS, the development of new and non-invasive methods for early diagnosis is of paramount significance. However, despite considerable efforts to establish noninvasive methods for diagnosing PCOS, reliable tests are still lacking. Most of the available studies focus on finding a single marker whose expression is significantly altered in patients with PCOS. As we have already mentioned, miRNAs are single-stranded non-coding RNA molecules involved in various biological processes and can be found both intracellularly and extracellularly, including body fluids [10]. Among their biological activities, they are considered both a marker and a regulating factor in PCOS [109]. Subsequently, follicular fluid and blood sample miRNA expression patterns were suggested as highly specific and reliable diagnostic biomarkers [119]. Table 4 presents the most relevant studies that identified miRNAs as diagnostic biomarkers and therapeutic targets, respectively.

Regarding the new biomarker investigations, the results are mixed but promising. An important meta-analysis of these datasets was performed by Deswal et al., identifying all relevant studies through May 2019 [132]. They revealed an initial set of 79 differentially expressed miRNAs across the 21 studies published, only three of which were reported in more than three studies. After meta-analysis, they reported miR-29a-5p and miR-320 as significant biomarkers for PCOS [132]. Another recent meta-analysis suggested that, compared with healthy controls, the expression of miR-93 was upregulated in PCOS patients ,while the expression of miR-320 was downregulated in PCOS patients but with significant heterogeneity [104]. Regarding miRNA diversity in PCOS patients, a study by Roth and coworkers demonstrated the expression of 235 miRNAs, with some miRNAs showing differential expression compared to control groups [56]. Furthermore, the expression profile of five extracellular miRNAs, including miR-let-7b, miR-29a, miR-30a, miR-140 and miR-320a, was investigated in the follicular fluid of 30 women with PCOS and 91 women with normal ovarian reserve [59]. The results showed that the combination of 3 miRNAs expressed in follicular fluid, including miR-30a, miR-140 and let-7b, could discriminate between PCOS and normal ovarian reserve. Therefore, miRNAs could also facilitate personalized medical care during IVF, and it could be hypothesized that extracellular miRNAs could potentially serve as novel biomarkers for PCOS diagnosis. Another study described the potential diagnostic magnitude of miR-93 in follicular fluid after it was somewhat increased in women with PCOS compared to controls [58]. As for the downregulated molecules, miR-29a, miR-24-3p and miR-574-3p were the most downregulated in women with PCOS compared to controls [113]. A combination panel of miR-let-7b, miR-30a and miR-140 expression demonstrated acceptable sensitivity and specificity in discriminating between PCOS and normal ovarian reserve, especially in IVF/ICSI [59,115]. Thus, this could be a novel biomarker that predicts outcomes and enables tailored medical care for patients with PCOS. Currently, evidence of molecular regulations that cause the differential expression of miRNAs is helpful to elucidate the pathogenesis of PCOS. However, many studies on miRNAs and PCOS in diagnostics were only partially consistent and provided contradictory results despite the promising data. The heterogeneity of PCOS probably employing a set of miRNAs rather than a single miRNA as biomarkers could improve prediction and accuracy in the diagnosis of PCOS.

Based on current guidelines, PCOS treatment strategies aim to relieve symptoms, improve prognosis and reduce the incidence of metabolic sequelae during life. Contemporary pharmacological approaches include oral contraceptives to regulate the menstrual cycle, ovulation inducers to restore fertility, oral insulin sensitizers to reduce insulin resistance and antiandrogens to prevent the progression of androgen-related symptoms that affect the quality of life [134]. One of the most efficient approaches is metformin use, which improves the main pathological PCOS features and can facilitate glucose transport by improving insulin sensitivity, lower free androgen levels and increasing fertility [135,136,137]. Recently, miRNAs have also attracted much interest as a potential target for the therapy of PCOS and are studied along with metformin use. Metformin administration has been associated with the altered expression of miR-26a in pancreatic cancer stem cell markers [129], miR-221 and miR-222 in patients with diabetes mellitus type 2 [131] and DICER1 in a novel therapeutic approach for age-related health problems [138]. A similar relationship, involving the altered expression of miR-33, miR-155-5p, miR-197, miR-6356, miR-1197-3p, miR-875-5P and miR-6763 has been observed in studies on glucagon-like peptide 1 agonist receptor agonist (GLP-1 RA) and dipeptidyl peptidase-4 (DPP-4) inhibitors [130,133]. These potential effects of miRNAs on insulin sensitivity might have a substantial role in improving PCOS-related symptoms by augmenting glucose transport and metabolism [139].

## 5. Conclusions

In summary, PCOS diagnosis and management is one of the most challenging issues for clinicians and health care professionals. In recent years, several promising studies have focused on the characterization and identification of different miRNAs. Some miRNAs associated with PCOS are abundant in the follicular fluid, serum, whole blood, theca and granulosa cells. They regulate follicular development and maturation, insulin signaling, glucose and lipid metabolism, and even steroid hormone synthesis. Considering all this, miRNAs could potentially be clinical biomarkers for the diagnosis of PCOS and a therapeutic target for the treatment of PCOS. An altered miRNA profile holds the potential for new methods to stratify PCOS patients and may contribute to and partially explain heterogeneity in PCOS women.

## Figures and Tables

**Table 1:** Characteristics of the included studies.

Author, Year	Study Type	Sample Size	Age (Years) PCOS/Controls	Main Results Dysregulated miRNAs (Upregulated/Downregulated)	Detected in Cell/Tissue
Cai et al., 2017 [28]	Case-control study	*N* = 50 (25 married women with PCOS and 25 controls)	N/A	Downregulated: miR-145	Granulosa cells
Chen et al., 2013 [29]	Case-control study	*N* = 41 (20 control and 21 PCOS)	27.46 ± 4.07/ 32.41 ± 6.61	Upregulated: miR-93, 133 and 223	Adipose tissue
Ding et al., 2015 [30]	Case-control study	Screening cohort (*N* = 18, 9 PCOS, 9 controls) Verification cohort (*N* = 18, 9 PCOS, 9 controls)	27.9 ± 4.3/ 28.7 ± 5.2	Upregulated: miR-5706, let-7i-3 pm, 4463, 3665 and 638 Downregulated: miR-124-3p, 128, 29a-3p and let-7c	Serum
Ebrahimi et al., 2018 [31]	Case-control study	*N* = 372 (180 with PCOS, 192 controls)	26.8 ± 5.5/ 27.0 ± 4.38	Upregulated: miR-146a	Whole blood
Eisenberg et al., 2017 [32]	Case-control study	*N* = 40 (7 normally ovulating, 15 normally ovulating with pure male infertility factor, and 18 with PCOS)	26.9 ± 4.3/ 26.8 ± 4.7	Upregulated: miR-200b and 429	Serum
Geng et al., 2019 [33]	Case-control study	*N* = 30 (15 married women with PCOS and 15 controls)	27.23 ± 1.83/ 28.53 ± 1.85	Upregulated: miR-99a	Granulosa cells
He et al., 2018 [34]	Case-control study	*N* = 123 (62 with PCOS, 61 controls)	28.27 ± 3.10/ 28.71 ± 2.46	Downregulated: miR-141 and 200c	Granulosa cells
Hosseini et al., 2017 [35]	Case-control study	*N* = 410 (205 with PCOS, 205 controls)	31.2 ± 5.5/ 28.5 ± 5.0	Upregulated: miR-146a and 222	Plasma
Hou et al., 2019 [36]	Case-control study	*N* = 28 (15 with PCOS, 13 controls)	29.60 ± 0.66/ 29.66 ± 0.82	Upregulated: miR-3188 and 3135b	Granulosa cells
Hu et al., 2020 [37]	Case-control study	N/A	N/A	Upregulated: miR-6087, 199a-5p, 1433p, 483-5p, 200a-3p, and 23b-3p	Follicular fluid
Huang et al., 2016 [38]	Case-control study	*N* = 36 (18 with PCOS, 18 controls)	32.6 ± 3.1/ 34.6 ± 2.2	Upregulated: miR-135b-5p, 152, 193a-3p, 194-5p, 196a-5p, 200b-3p, 423-3p, 454-3p, 455-5p, 4659a-3p, 509–3-5p, 509-3p, 513b-5p, 652-5p, 95, 1273e	Cumulus cells
Jiang et al., 2016 [39]	Case-control study	*N* = 175 (65 with PCOS and IGM; 65 with PCOS without IGM; 45 healthy controls)	27.16 ± 3.56/ 27.98 ± 3.66	Upregulated: miR-122, 194, and 193b Downregulated: miR-199b-5p	Serum
Li et al., 2019 [40]	Case-control study	*N* = 78 (46 with PCOS, 32 controls)	29.21 ± 4.78/ 29.43 ± 3.82	Upregulated: miR-33b and 142 Downregulated: miR-423	Granulosa cells
Lin et al., 2015 [41]	Case-control study	*N* = 18 (10 with PCOS, 8 controls)	28.80 ± 3.97/ 32.00 ± 2.16	Downregulated: miR-19b, 92a, 92b, 141, and 200a	Ovarian theca internal tissues
Linlin Jiang, 2015 [42]	Case-control study	*N* = 24 (16 with PCOS, 8 controls)	29.69 ± 2.39/ 31.75 ± 4.40	Upregulated: miR-93, 107	Granulosa cells
Liu et al., 2015 [43]	Case-control study	*N* = 20 (10 with PCOS, 10 controls)	27.4 ± 2.6/ 29.4 ± 3.0	Upregulated: miR-513a-3p, 508-3p, 513b, 514, 509-5p, 513c, 144, 510, 509-3p and 508-5p Downregulated: miR-151-3p, 720, 615-3p, 127-3p, 455-3p, 342-3p and 654-3p	Cumulus cells
Liyan Jiang et al., 2015 [44]	Case-control study	N/A	N/A	Upregulated: miR21,222,16,19a,30c, 146a, 24 and 186	Serum
Long et al., 2014 [45]	Multistage restrospective, nested case-control study	*N* = 136 (68 with PCOS, 68 controls)	26.6 ± 2.8/ 27.9 ± 3.4	Upregulated: miR-222, 146a and 30c	Serum
Luo et al., 2019 [46]	Case-control study	*N* = 12 (4 with POR, 4 with PCOS, 4 controls)	PCOS (27.00 ± 3.26) POR (37.00 ± 3.16) Controls (29.00 ± 3.22)	Upregulated: miR-23a	Granulosa cells
Mao et al., 2018 [47]	Case-control study	*N* = 69 (43 with PCOS, 26 controls)	30.2 ± 2.8/ 31.1 ± 2.1	Downregulated: miR-126-5p and 29a-5p	Granulosa cells
McAllister et al., 2019 [48]	Case-control study	*N* = 14 (7 with PCOS, 7 controls)	N/A	Upregulated: miR-100-5p, 99b-5p, 1271-5p, 409-5p, 744, 410-3p, 127-3p, 654-5p, 494-3p, 1301-3p, 502-3p, 501-3p and 1293 Downregulated: miR-125a-3p,148b-5p, 195-5p,130b-3p and 4542a-5p	Ovarian theca cells
McCallie et al., 2010 [49]	Descriptive study	*N* = 44	N/A	Downregulated: miR-let-7a, 19a, 19b, 24, 92, and 93	Blastocysts
Murri et al., 2013 [50]	Case-control study	*N* = 36 (12 with PCOS, 12 healthy women, 12 men)	27 ± 4/ 29 ± 3	Upregulated: miR-21,27b, 103 and 155	Whole blood
Murri et al., 2018 [51]	Case-control study	*N* = 35 (12 with PCOS, 11 healthy women, 12 men)	27 ± 4/ 28 ± 3	Upregulated: miR-34c-5p and 548d-3p Downregulated: miR-26a-5p, 30c-5p, 107 and 199a-3p	Serum
Naji et al., 2017 [52]	Case-control study	*N* = 66 (19 with normoandrogenic PCOS, 22 with hyperandrogenic PCOS, 25 controls)	28.89 ± 1.07/ 28.24 ± 0.82	Upregulated in granulosa cells: miR-93 Downregulated in follicular fluid: miR-93 and 21	Follicular fluid, granulosa cells
Naji et al., 2018 [53]	Case-control study	*N* = 41 (20 with PCOS, 21 controls)	29.25 ± 0.84/ 28.42 ± 0.91	Upregulated in follicular fluid: miR-182 Downregulated in granulosa-lutein cells: miR-145 and 182	Serum, granulosa-lutein cells, follicular fluid
Nanda et al., 2020 [54]	Case-control study	*N* = 40 (20 with PCOS, 20 controls)	28.35 ± 7.45/ 25.15 ± 4.12	Upregulated: miR-122, 194, and 193b Downregulated: miR-199b-5p	Serum
Rashad et al., 2019 [55]	Case-control study	*N* = 100 (60 with PCOS, 40 controls)	N/A	Downregulated: miR-320	Serum
Roth et al., 2014 [56]	Case-control study	N/A	33.1 ± 4.4/ 27.1 ± 3.6	Upregulated: miR-32, 34c, 135a, 18b, and 9	Follicular fluid
Sang et al., 2013 [57]	Case-control study	*N* = 44 (22 with PCOS, 22 controls)	29.09 ± 0.70/ 30.83 ± 0.90	Downregulated: miR-132 and 320	Follicular fluid
Sathyapalan et al., 2015 [58]	Case-control study	*N* = 49 (25 with PCOS, 24 controls)	32.1 ± 9.0/ 32.2 ± 7.7	Upregulated: miR-93 and 223	Plasma
Scalici et al., 2016 [59]	Case-control study	*N* = 121 (30 with PCOS, 91 controls)	Mean age for cohort 33.7 ± 4.5	Upregulated: miR-30a Downregulated: miR-140 and let-7b	Follicular fluid
Shi et al., 2015 [60]	Case-control study	*N* = 48 (24 with PCOS, 24 controls)	28.3 ± 3.3/ 28.5 ± 3.6	Downregulated: miR-483–5p and 486–5p	Cumulus cells
Song et al., 2015 [61]	Case-control study	*N* = 134 (67 with PCOS, 67 controls)	26.7 ± 2.7/ 27.6 ± 3.3	Downregulated: miR-592,124-3p, 128, 29-3p, 16, 106b, 19a, 24, 186, let-7c and 1228	Serum
Song et al., 2016 [62]	Case-control study	*N* = 42 (21 with PCOS, 21 controls) with preceded pilot study (*N* = 17)	23 ± 4/ 24 ± 6	Downregulated: miR-4522, 324-3p, and 6767-5p	Serum
Song et al., 2019 [63]	Case-control study	*N* = 83 (63 with PCOS, 20 controls)	28.21 ± 2.78/ 27.43 ± 3.62	Upregulated: miR-186 and 135a	Granulosa cells
Sørensen et al., 2016 [64]	Case-control study	*N* = 70 49 PCOS women (19 of which were hyperandrogenic and 30 normo-androgenic) and 21 healthy matched women	28.1 ± 4.3/ 27.8 ± 3.8	Upregulated: miR-518f-3p, Downregulated: miR-24-3p, -29a, -151-3p, and -574-3p	Follicular fluid
Sørensen et al., 2016 [65]	Case-control study	*N* = 62 42 PCOS patients and 20 Controls	27.0 ± 7.5/ 27.0 ± 6.3	Upregulated: miR-485-3p, miR-1290, and miR-7-1-3p Downregulated: miR-21-3p, miR-139-3p, miR -572, miR-361-5p, miR-143-3p, miR-345-5p, miR-1276, and miR-22-5p	Follicular fluid
Wang et al., 2018 [66]	Case-control study	*N* = 30 17 PCOS patients and 13 controls	28.7 ± 0.7/ 30.0 ± 0.7	Upregulated: miR-27a-3p	Granulosa cells
Wang et al., 2019 [67]	Case-control study	*N* = 45 24 PCOS patients and 21 controls	28.7 ± 0.8/ 29.6 ± 1.0	Upregulated: miR-3188 and 3135b	Granulosa cells
Wu et al., 2014 [68]	Case-control study	*N* = 31 16 women with PCOS (8 with and 8 without IR) and 15 non-PCOS (9 with and 6 without IR).	32.33 ± 5.03/ 25.49 ± 5.56	Upregulated: miR-93, and 25	Adipose tissue
Xiang et al., 2016 [69]	Case-control study	*N* = 40 (20 with PCOS, 20 controls)	27.3 ± 2.5/ 28.2 ± 3.7	Downregulated: miR-483	Ovarian cortex
Xiong et al., 2017 [70]	Case-control study	*N* = 48 (18 with PCOS, 30 controls)	N/A	Downregulated: miR-23a and 23b	Serum
Xu et al., 2015 [71]	Case-control study	*N* = 41 (21 with PCOS, 20 controls)	N/A	Upregulated: miR-423-3p, 3651, 3653, 151b, 1273 g-3p, 590-5p, 3648, 7845-5p, 27a-5p, 1275, 483-3p, 7-5p, 483-5p, 10a-5p, 184, 619-5p, 513b-5p, 1307-5p, 4516, 1307-3p, 514b-5p Downregulated: miR-3529-3p, 7974, 3065-5p, 214-3p, 200a-3p, 203a, 4732-5p, 423-5p, 3184-5p, 548n, 221-3p, 149-5p, 1298-5p, 193a-3p, 365a-3p, 219a-1-3p, 550b-2-5p, 144-5p, 660-5p, 548e-3p, 652-3p, 222-3p,506-5p, 193a-5p, 210-5p, 365b-5p, 330-3p, 223-3p, 186-5p, 185-5p, 92b-3p, 199b-3p, 766-5p, 15b-3p, 339-5p, 3960, 766-3p, let-7a-3p	Cumulus granulosa cells
Xue et al., 2018 [72]	Case-control study	*N* = 6 (3 with PCOS, 3 controls)	N/A	Upregulated: miR-200a-3p, 10b-3p, 200b-3p,29c-3p, 99a-3p and 125a-5p Downregulated: miR-105-3p	Follicular fluid
Yao et al., 2018 [73]	Prospective, observational study	Female Sprague–Dawley rats (23 days old)	N/A	Downregulated: miR-335-5p	Follicular fluid
Yao et al., 2018 [74]	Case-control study	*N* = 106 (55 with PCOS, 51 controls)	28.13 ± 0.41/ 27.37 ± 0.46	Downregulated: miR-335-5p	Follicular fluid
Yin et al., 2014 [75]	Prospective, observational study	Mice ovaries	N/A	Upregulated: miR-320 and miR-383	Follicular fluid, granulosa cells
Zhang et al., 2017 [76]	Case-control study	*N* = 33 (21 with PCOS, 12 controls)	N/A	Downregulated: miR-320a	Cumulus cells
Zhang et al., 2018 [77]	Case-control study	*N* = 40 (20 with PCOS, 20 controls)	N/A	Upregulated: miR-873-5p	Follicular fluid
Zhao et al., 2015 [78]	Multistage retrospective nested case-control study	*N* = 384 patients with PCOS; 30 with developed OHSS after IVF/ICSI and 70 controls	27.93 ± 3.84/ 27.70 ± 3.44	Upregulated: miR-146a, 30c and 191 Downregulated: miR-16, 223, 212, 451 and 92a	Serum
Zhong et al., 2018 [79]	Case-control study	*N* = 28 (18 with PCOS, 10 controls)	N/A	Downregulated: miR-19b	Granulosa cells, ovarian cortex

Abbreviations: IGM—impaired glucose metabolism; IR—insulin resistance; miR—microRNA; N/A—not available; OHSS—ovarian hyperstimulation syndrome; PCOS—polycystic ovarian syndrome; POR—poor ovarian response.

**Table 2:** Summary of the reported biological role of the most notably dysregulated miRNAs in terms of hormonal homeostasis.

miRNA	Species	Reported Biological Role	
miR-9	Human	Inhibits testosterone release [56]	
miR-18b	Human	Inhibits testosterone and estradiol release [56] Promotes progesterone release [56]	
miR-20a	Human	Postively correlated with hyperandrogenism (increase of either total testosterone, free testosterone, DHEAS or androstenedione) [38]	
miR-21	Human Rat	Anti-apoptotic properties; involved in oocyte maturation [53]	
miR-24	Human	Inverse correlation with insulin, LH, testosterone and the LH:FSH ratio [44]	
miR-27b	Human	Positively correlated with testosterone; increased expression in PCOS [50]	
miR-29a	Human	Inveresely associated with LH and insulin; positively correlated with DHEA and androstendione [54,80]	
miR-30c	Human Rat	Increased expression after FSH exposure (positive association) [44]	
miR-34b-3p	Human	Negative association with with hyperandrogenism (either total testosterone, free testosterone, DHEAS or androstenedione) [51]	
miR-103	Human	Promotes progesterone release and inhibits estradiol release [50]	
miR-107	Human	Increases testosterone release [51]	
miR-132	Human Rat	Increases estradiol secretion [57] Reduces progesterone and testosterone release [57]	
miR-135a	Human	Reduces progesterone and testosterone release [63,81]	
miR-139	Human	Negative association with with hyperandrogenism (either total testosterone, free testosterone, DHEAS or androstenedione) [65]	
miR-146a	Human	Reduces progesterone, estradiol and testosterone release [31]	
miR-151	Human	Postively associated with total testosterone and free testosterone [43]	
miR-155	Human	Inhibits testosterone release [50]	
miR-222	Human	Increases estradiol secretion [35]	
miR-224	Human Mouse	Induces granulosa cells proliferation [82] Increases estradiol release [82]	
miR-320	Human	Increases testosterone release [75,76]	
miR-361	Human	Positive correlation with SHBG levels [65]	
miR-383	Human	Increases estradiol secretion [75]	
miR-433	Human	Positive correlation with SHBG levels [37]	
miR-518	Human	Positively correlated with total and free testosterone, DHEA-S and androstendione [64]	
miR-1225	Human	Negatively associated with serum insulin and HOMA-IR [83]	
miR-1290	Human	Postively correlated with hyperandrogenism (increase of either total testosterone, free testosterone, DHEAS or androstenedione) [65]	

**Table 3:** Risk of bias analysis (according to the Newcastle–Ottawa scale) of studies included evaluating the translational role of miRNA in PCOS. Each “*” indicates one point on the Newcastle–Ottawa scale.

Study	Selection	Comparability	Outcome	Overall
Cai et al., 2017 [28]	***	**	**	7
Chen et al., 2013 [29]	***	**	***	8
Ding et al., 2015 [30]	***	***	***	9
Ebrahimi et al., 2018 [31]	***	*	**	6
Eisenberg et al., 2017 [32]	***	**	***	8
Geng et al., 2019 [33]	***	**	**	7
He et al., 2018 [34]	***	***	**	8
Hosseini et al., 2017 [35]	**	**	**	6
Hou et al., 2019 [36]	***	**	**	7
Hu et al., 2020 [37]	***	*	***	7
Huang et al., 2016 [38]	***	**	***	8
Jiang et al., 2016 [39]	***	***	***	9
Li et al., 2019 [40]	***	**	**	7
Lin et al., 2015 [41]	***	***	**	8
Linlin Jiang, 2015 [42]	**	**	***	7
Liu et al., 2015 [43]	***	**	***	8
Liyan Jiang et al., 2015 [44]	**	**	**	6
Long et al., 2014 [45]	***	***	***	9
Luo et al., 2019 [46]	**	**	***	7
Mao et al., 2018 [47]	***	***	**	8
McAllister et al., 2019 [48]	**	**	***	7
McCallie et al., 2010 [49]	**	**	***	7
Murri et al., 2013 [50]	***	***	**	8
Murri et al., 2018 [51]	***	***	***	9
Naji et al., 2017 [52]	***	***	***	9
Naji et al., 2018 [53]	***	***	***	9
Nanda et al., 2020 [54]	**	***	**	7
Rashad et al., 2019 [55]	**	**	**	6
Roth et al., 2014 [56]	**	**	***	7
Sang et al., 2013 [57]	***	***	**	8
Sathyapalan et al., 2015 [58]	***	***	**	8
Scalici et al., 2016 [59]	***	***	***	9
Shi et al., 2015 [60]	***	**	**	7
Song et al., 2015 [61]	***	***	**	8
Song et al., 2016 [62]	***	***	**	8
Song et al., 2019 [63]	***	***	**	8
Sørensen et al., 2016 [64]	***	***	***	9
Sørensen et al., 2016 [65]	***	***	***	9
Wang et al., 2018 [66]	***	***	**	8
Wang et al., 2019 [67]	***	***	**	8
Wu et al., 2014 [68]	***	***	**	8
Xiang et al., 2016 [69]	***	**	**	7
Xiong et al., 2017 [70]	***	**	**	7
Xu et al., 2015 [71]	***	**	***	8
Xue et al., 2018 [72]	**	**	**	6
Yao et al., 2018 [73]	**	**	**	6
Yao et al., 2018 [74]	***	**	**	7
Yin et al., 2014 [75]	**	***	**	7
Zhang et al., 2017 [76]	**	**	***	7
Zhang et al., 2018 [77]	**	**	***	7
Zhao et al., 2015 [78]	***	***	***	9
Zhong et al., 2018 [79]	**	**	***	7

* The Newcastle–Ottawa scale contains 8 items within 3 domains (selection, comparability, outcome); the total maximum score is 9. A study with a score above 7 points indicates a good-quality study, 4–6 indicates a high risk of bias, and a score under 4 points indicates a very high risk of bias.

**Table 4:** A summary of the most relevant studies that identified miRNAs as diagnostic biomarkers and therapeutic targets.

Study	miRNAs as Diagnostic Biomarkers	miRNAs as Therapeutic Targets
Ali et al. [129]	N/A	miR-26a
Capuani et al. [130]	N/A	miR132, miR-212, miR-338, miR-758, miR34a, miR-21, miR-200b, miR-200c
Coleman et al. [131]	N/A	miR-221 and miR-222
Deswal et al. [132]	miR-29a-5p and miR-320	N/A
Mu et al. [104]	miR-93 and miR-320	N/A
Radbakhsh et al. [133]	N/A	miR-33, miR-155-5p, miR-197, miR-6356, miR-1197-3p, miR-875-5P and miR-6763
Sathyapalan et al. [58]	miR-93	N/A
Scalici et al. [59]	miR-let-7b, miR-30a and miR-140	N/A

miRNA—microRNA; N/A—not available.

## Data Availability

Not applicable.
